# Venipuncture Induced Complex Regional Pain Syndrome Presenting as Inflammatory Arthritis

**DOI:** 10.1155/2016/8081401

**Published:** 2016-11-07

**Authors:** Punit Pruthi, Pramod Arora, Manoj Mittal, Anugrah Nair, Waqia Sultana

**Affiliations:** ^1^Department of Internal Medicine and Rheumatology, Asian Institute of Medical Sciences, Faridabad, India; ^2^Department of Nuclear Medicine, Asian Institute of Medical Sciences, Faridabad, India; ^3^Department of Radiodiagnosis, Asian Institute of Medical Sciences, Faridabad, India

## Abstract

Venipuncture is one of the most commonly done medical procedures. We report a unique case of a 23-year-old young male who presented with features suggestive of inflammatory arthritis. The symptoms, which initially started on the right side, also involved the other side after a few weeks. Although the patient's symptoms and signs were simulating inflammatory arthritis, he had atypical features like poor response to anti-inflammatory medicines and normal laboratory parameters. His musculoskeletal ultrasonography was also not suggestive of arthritis. His history was reviewed and on direct questioning he revealed a history of venipuncture for blood sample withdrawal, done from right antecubital region for routine health check on the day prior to the onset of symptoms. Complex regional pain syndrome was suspected and triple-phase radioisotope bone scan was done which was highly suggestive of this diagnosis. The patient was managed with multidimensional approach and responded very well to the treatment. Complex regional pain syndrome is usually not thought of in the initial differential diagnosis of inflammatory arthritis. In this report we highlight the need to elicit the often overlooked history of trivial trauma like venipuncture, especially in atypical cases of arthritis. Also the role of newer diagnostic modalities in such cases is emphasized.

## 1. Introduction

Complex regional pain syndrome (CRPS) is a pain disorder characterized by spontaneous pain, usually triggered by a noxious stimulus like trauma which may be trivial, immobilization, and postacute illnesses like myocardial infarction or stroke. In around 10% of cases no inciting cause is identified [1]. CRPS was traditionally divided into type 1 and type 2, depending on the absence or presence of a preceding peripheral nerve injury [1]. The necessity of distinguishing these two forms has been questioned, since in most cases nerve involvement cannot be definitively excluded and the forms are clinically identical [2, 3].

The diagnosis is made as per the Budapest criteria, according to which patients should have continuing pain disproportionate to the inciting event [4]. Symptoms and signs are grouped under four categories: sensory, vasomotor, sudomotor/oedema, and motor/trophic. CRPS is diagnosed if the patient has at least one sign in two or more categories and at least one symptom in three or more categories, in the absence of alternative explanations [4].

Venipuncture is a routinely done procedure and is usually forgotten in history elicitation both by the physician and by the patient. We report a case of a young male who developed joint symptoms one day after blood sample withdrawal.

## 2. Case Presentation

A 23-year-old male patient from India, without any past medical history, presented with pain and swelling in small joints of right hand with morning stiffness lasting for more than one hour along with feeling of warmth of the hands. The symptoms were of six-week duration, and within the week prior to being seen he also developed similar pain in the joints of his left hand. The pain was constantly present with exacerbation on touch and on movement at joints of hands. There were no constitutional or systemic features and he denied any history of trauma.

**Figure 1:**
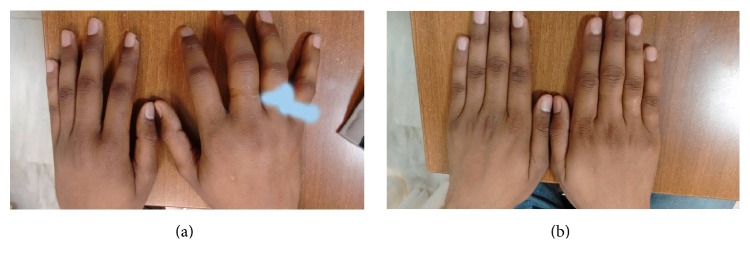
(a) Diffuse swelling of the right hand, more so around the joints. Similar features with lesser severity seen on left hand. His rings (masked by white colour) on the fourth and fifth fingers of the right hand got stuck due to swelling. (b) Complete normalization of swelling after two months of treatment.

On examination there was marked tenderness over and around small joints of both hands, particularly on right side. There was diffuse swelling of the right hand which was more over and around the joints, and also the hand was slightly warm to touch (Figure 1(a)). There was decreased range of motion at the wrist and small joints of hand. Similar findings with lesser severity were also present on the left hand. Apart from marked joint tenderness there was no significant dysaesthesia or sensory loss over the hands. He reported difficulty in performing routine activities with his hands due to severe pain.

Prior to his presentation to us his family physician had him tested for rheumatoid factor and anticyclic citrullinated peptide antibodies, and both were negative. He had also received multiple analgesics and anti-inflammatory medicines without any significant relief.

On further evaluation, his laboratory parameters including haemoglobin, blood counts, kidney and liver function tests, and urine examination were normal. His erythrocyte sedimentation rate was 5 mm/hour (normal range 0–10 mm/hour) and C-reactive protein was 1.8 mg/dL (normal range 0–0.75 mg/dL). Plain radiographs of the chest and both hands (including wrists) were normal.

**Figure 2:**
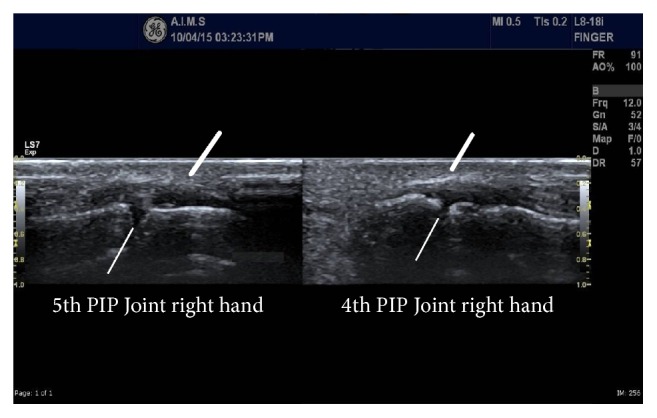
B-mode high resolution ultrasonography image (longitudinal section) of proximal interphalangeal joints showing diffuse dermal and subcutaneous oedema (thick lines) and normal joint space with no evidence of synovitis (thin lines).

Musculoskeletal ultrasonography was done which revealed diffuse subcutaneous oedema of both hands (more on the right side) without any evidence of synovitis (Figure 2). In view of no concrete evidence of inflammatory arthritis in his laboratory and radiological investigations and poor response to anti-inflammatory medicines, his history was retaken and on direct questioning he revealed the history of venipuncture for blood sampling from right antecubital region, done for his routine health check one day prior to the onset of symptoms. Venipuncture was done skillfully and he denied any history of sudden electric current like sensation, pain, tingling, weakness, or loss of sensation after the procedure.

A possibility of complex regional pain syndrome was strongly considered as the patient fulfilled the Budapest diagnostic criteria; he had continuing pain disproportionate to the inciting event, hyperalgesia, vasomotor changes in the form of rise in temperature of the hands, sudomotor changes in the form of oedema of hands, and motor finding of decreased range of motion at wrist and small joints of hands. No other condition could be found that could have explained the clinical scenario.

**Figure 3:**
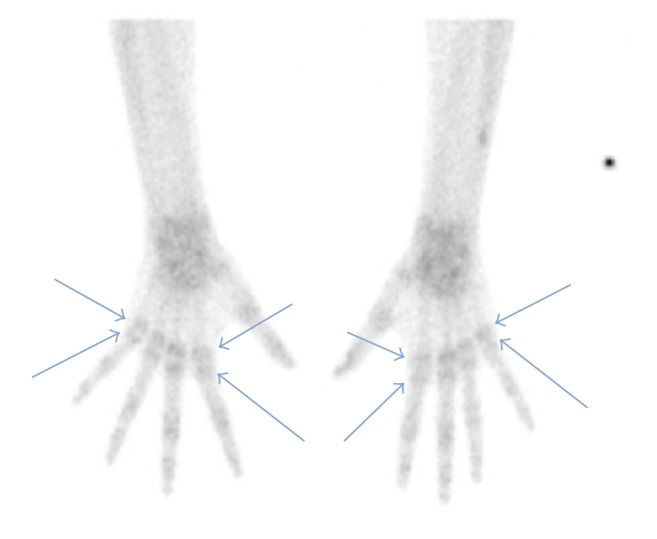
Triple-phase radioisotope bone scan (delayed phase) showing increased periarticular tracer uptake (arrows) with sparing of the joint space, more evident in metacarpophalangeal joints of both hands, and also diffusely increased tracer uptake over both wrists.

In order to further strengthen the diagnosis a triple-phase radioisotope bone scan was done which revealed findings highly suggestive of CRPS in both upper limbs (Figure 3). The bone site of increased uptake was exactly the site that Sudeck first described periarticular demineralization [5].

Patient was treated with pregabalin, a single intramuscular depot preparation of 80 mg methylprednisolone, physiotherapy, and counseling. His symptoms gradually abated within two months of therapy and swelling of the hands subsided (Figure 1(b)). His pregabalin was tapered off without any recurrence in the next one year of follow-up.

## 3. Discussion

The clinical course of CRPS was traditionally divided into acute, dystrophy, and atrophy stages based on duration of illness and symptomatology [1]. The evolution of CRPS into these three stages has been questioned and now stages have been removed from the taxonomy and two types, acute/warm and chronic/cold, prevail often without temporal change [2, 3, 6]. Patients with warm CRPS tend to have pain of less than six months duration whereas patients with cold presentation usually have pain for more than 20 months [6]. As observed in our case, acute CRPS may mimic various inflammatory disorders including arthritis [6]. It is relatively easy to treat when diagnosed early and has a better prognosis as compared to chronic CRPS which is more likely to result in dystonia, atrophy, and dystrophic changes [2, 3]. A fulminant acute form if not treated may progress to the chronic form within two months [2, 6]. The disease may involve the other limbs also in due course of time as seen in our patient [1].

CRPS secondary to venipuncture has been described previously [7, 8]. Nerve injury may occur due to direct needle trauma to the nerve or due to extravasation of intravenous fluid or blood into the plain of the nerves beneath the veins. Although anatomically superficial veins and cutaneous nerves are in close proximity, venipuncture induced nerve injuries are rare [8]. So other mechanisms like central and peripheral sensitization, inflammation, sympathetic dysregulation, and microvascular dysfunction play major roles in the pathogenesis of CRPS [1, 7]. Antineuronal antibodies have been found in patients of CRPS; thus autoimmunity may be another mechanism contributing to the pathogenesis of CRPS [9].

Treatment of CRPS is multidimensional depending on the stage of presentation. Acute CRPS is usually treated with physiotherapy, psychotherapy, and drug therapy which may include tricyclic antidepressants, antiepileptics, bisphosphonates, anaesthetic agents, and calcitonin and free radical scavengers, such as N-acetylcysteine [1, 2]. Steroids and intravenous immunoglobulin have been used with varying success [9]. Progression to the chronic stage significantly worsens the prognosis. In refractory cases apart from the modalities used for the acute CRPS, transcutaneous electrical nerve stimulation, spinal cord stimulation, regional sympathetic blockades, surgical sympathectomy, and occupational therapy have been used with limited success [2, 7, 10].

Early symptoms of CRPS may closely mimic inflammatory arthritis and it should be actively considered in the differential diagnosis, especially in seronegative and atypical presentations of arthritis. Although CRPS is a clinical diagnosis, recent advances in musculoskeletal ultrasonography and radionuclear imaging techniques may help to resolve this task with the admonition that both techniques have reduced specificity with time [11]. However, the need for meticulous history taking and physical examination cannot be ignored.
